# Supplementary material for: BCGNet: an AI model trained on 600 K hours of sleep data for a novel under-pillow contactless monitoring device
Source: NPJ Digit Med. 2026 Jul 3;9:813. doi: 10.1038/s41746-026-02885-y (PMC13696710; doi:10.1038/s41746-026-02885-y)
Supplement: Supplementary file 1 [file 41746_2026_2885_MOESM1_ESM.pdf]

The authors provide this Supplement to give readers additional information about their work.

Supplementary results

Detailed Characteristics of Chinese Cohorts

Supplementary Table 1: PSG-derived sleep staging characteristics of the Chinese cohorts.

Dataset	Wake	N1	N2	N3	REM
**PSG Cohorts (Pre-training)**					
BTH	0.16 ± 0.14	0.10 ± 0.09	0.50 ± 0.16	0.09 ± 0.11	0.16 ± 0.07
SSPH	0.18 ± 0.15	0.15 ± 0.12	0.44 ± 0.14	0.13 ± 0.10	0.10 ± 0.06
IMMHC	0.23 ± 0.16	0.15 ± 0.09	0.49 ± 0.16	0.03 ± 0.05	0.11 ± 0.07
**BCG Cohorts**					
SSPH‡	0.22 ± 0.13	0.15 ± 0.11	0.32 ± 0.11	0.11 ± 0.09	0.14 ± 0.06
SSPH§	0.26 ± 0.15	0.13 ± 0.08	0.34 ± 0.10	0.10 ± 0.09	0.13 ± 0.05
WCH	0.27 ± 0.12	0.21 ± 0.11	0.37 ± 0.12	0.01 ± 0.03	0.14 ± 0.06
SAHSU	0.28 ± 0.16	0.12 ± 0.09	0.36 ± 0.11	0.11 ± 0.07	0.13 ± 0.06

Note: All sleep stage proportions are based on expert-scored PSG annotations. The term "BCG cohorts" indicates cohorts used for BCGNet fine-tuning or validation, not BCG-derived sleep staging. Values represent the proportion of total sleep time (mean ± standard deviation). ‡SSPH set used for fine-tuning. §SSPH set used for internal validation. WCH and SAHSU served as external validation cohorts.

Supplementary Table 2: Sleep apnea severity distribution.

Dataset	AHI3%	Healthy	Mild	Moderate	Severe	Total*
**PSG Cohorts (Pre-training)**						
BTH	24.65 ± 27.16	2135	1021	879	1987	6022
SSPH	32.98 ± 28.12	1426	1192	1126	3243	6987
IMMHC	12.69 ± 18.28	794	463	222	201	1680
**BCG Cohorts**						
SSPH‡	33.80 ± 27.50	108	131	111	358	708
SSPH§	21.45 ± 19.02	35	39	39	40	153
WCH	23.85 ± 22.65	124	175	116	176	591
SAHSU	25.05 ± 21.30	9	8	6	18	41

Note: All AHI values and severity classifications are based on expert-scored PSG annotations. The term "BCG cohorts" indicates cohorts used for BCGNet fine-tuning or validation, not BCG-derived AHI% values. AHI values are presented as mean ± standard deviation. Severity is classified based on AHI3%: Healthy (< 5), Mild ($5 - 15$), Moderate ($15 - 30$), Severe (≥ 30). ‡SSPH set used for fine-tuning. §SSPH set used for internal validation. *Total counts in this table represent subjects with complete valid AHI labels; these numbers are slightly lower than the total recorded subjects listed in the main text Table 1 due to missing or incomplete AHI annotation for a small subset of recordings.

Supplementary Table 1 summarizes the sleep architecture (stage proportions) across all Chinese cohorts, highlighting the distinct reduced N3 sleep in psychiatric cohorts (IMMHC, WCH). Supplementary Table 2 details the overall AHI statistics and the granular distribution of subjects across severity levels, confirming that our datasets cover a broad spectrum of disease severity suitable for robust validation.

Comprehensive performance of BCGNet in sleep staging

To further characterize the sleep staging capabilities of BCGNet, we evaluated its performance on both 3-class (Wake, NREM, REM) and 5-class (Wake, N1, N2, N3, REM) classifications across three independent, multi-center cohorts (SSPH, WCH, and SAHSU). The results, detailed in Supplementary Figure 1 and 2, demonstrate the BCGNet's robust and highly accurate performance. For the 3-class task, BCGNet achieved outstanding overall accuracies of 92.2%, 91.9%, and 87.5%, with corresponding

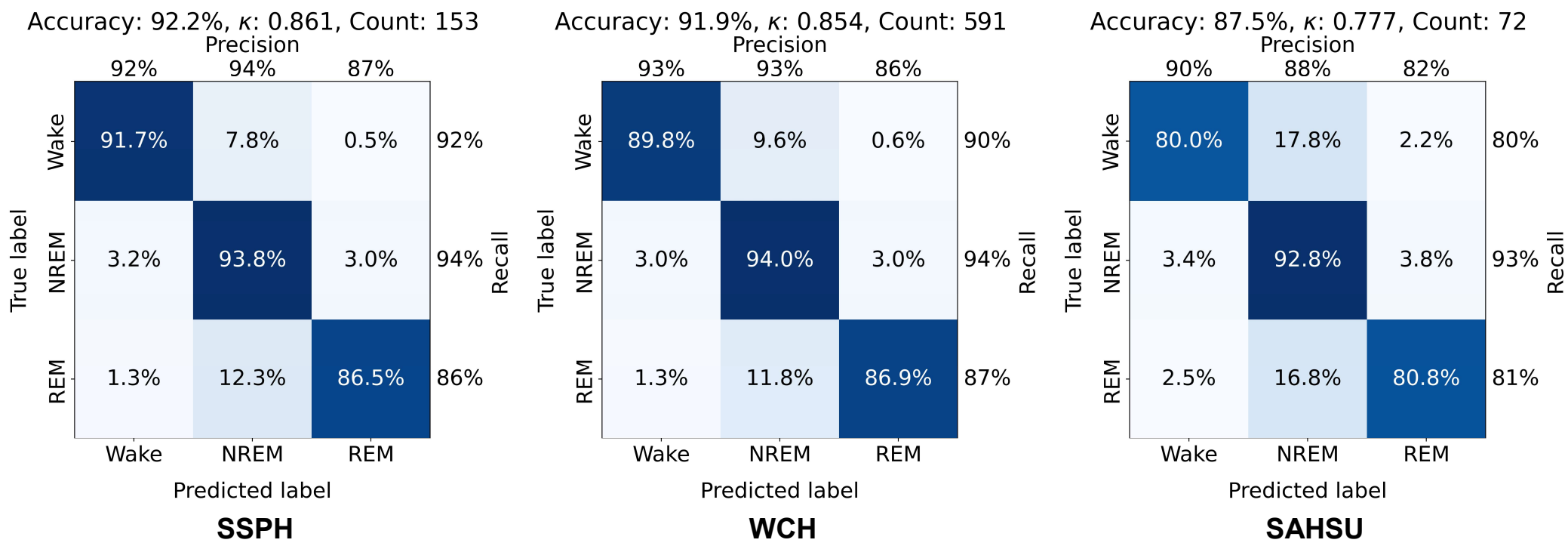

Supplementary Figure 1: Performance of BCGNet for 3-class sleep staging across multi-center cohorts. Performance is reported for the internal validation cohort (SSPH), and the external validation cohorts (WCH and SAHSU).

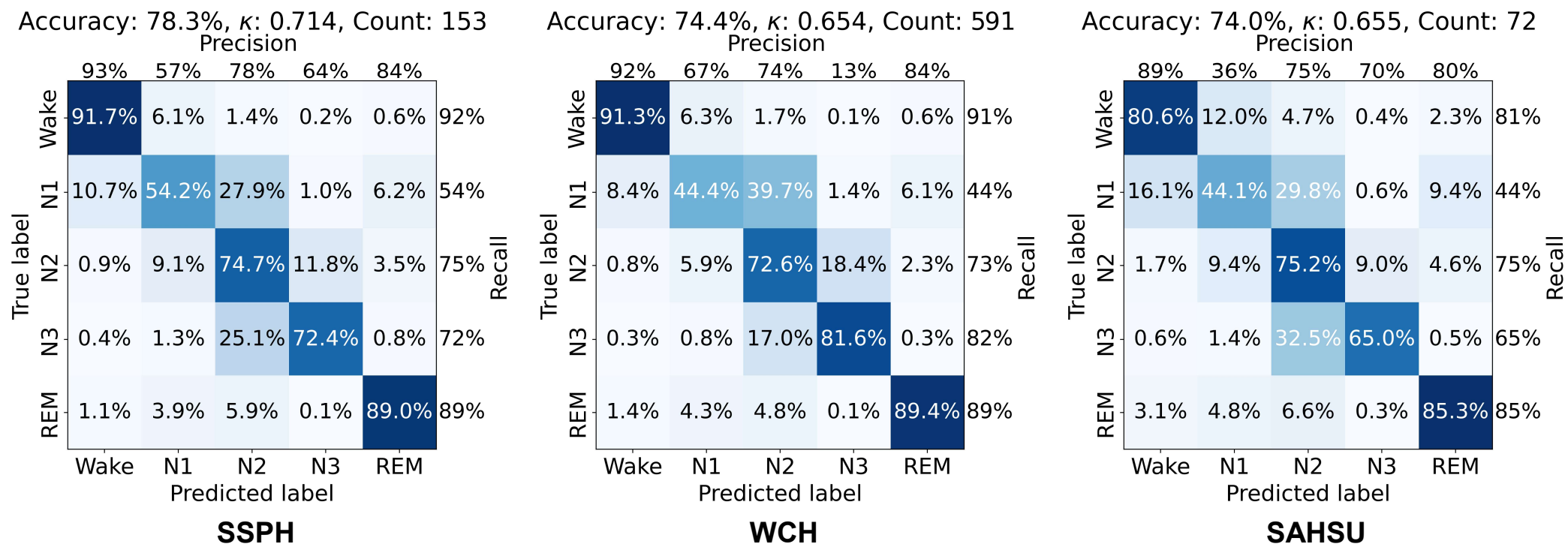

Supplementary Figure 2: Performance of BCGNet for 5-class sleep staging across multi-center cohorts. Performance is reported for the internal validation cohort (SSPH), and the external validation cohorts (WCH and SAHSU).

Cohen's κ of 0.861, 0.854, and 0.777, respectively. Moreover, the results on the external cohorts indicate a very high level of agreement (e.g., Cohen's κ of 0.854 in the WCH) with gold-standard PSG scoring and underscore the BCGNet's reliability in distinguishing fundamental sleep stages.

In the more granular and challenging 5-class staging task, BCGNet continued to exhibit strong performance, attaining accuracies of 78.3%, 74.4%, and 74.0% ($\kappa = 0.714$, 0.654, and 0.655) across the SSPH, WCH, and SAHSU cohorts. The confusion matrix of the external cohorts (Accuracy: 74.3%, κ: 0.655) reveals that the BCGNet excels at identifying clinically significant stages, such as deep sleep (N3) and REM sleep. While minor confusion exists between adjacent, lighter sleep stages (e.g., N1 and N2), a well-known challenge even for human experts, the overall high performance confirms BCGNet's accuracy for detailed sleep architecture analysis across diverse populations.

AHI Estimation Accuracy

Supplementary Table 3: **Mean Absolute Error (MAE) of AHI estimation across validation cohorts.**

	SSPH	WCH	SAHSU
**MAE (95% CI)**	3.952 (3.427, 4.573)	4.801 (4.393, 5.208)	4.480 (3.013, 6.213)

Note: MAE values are reported with 95% confidence intervals (CI) in parentheses.

To further quantify the precision of our model against the gold-standard PSG, we calculated the Mean Absolute Error (MAE) for AHI estimation across all external validation cohorts (see Table 3). The model achieved a low MAE ranging from 3.95 to 4.80 across the three centers. Specifically, the

MAE was 3.952 (95% CI: 3.427–4.573) for the SSPH cohort, 4.801 (95% CI: 4.393–5.208) for the WCH cohort, and 4.480 (95% CI: 3.013–6.213) for the SAHSU cohort.

Considering that the standard clinical cutoff for diagnosing mild sleep apnea is an AHI of 5 events/hour, an average error of less than 5 events/hour demonstrates that BCGNet provides highly reliable AHI estimates.

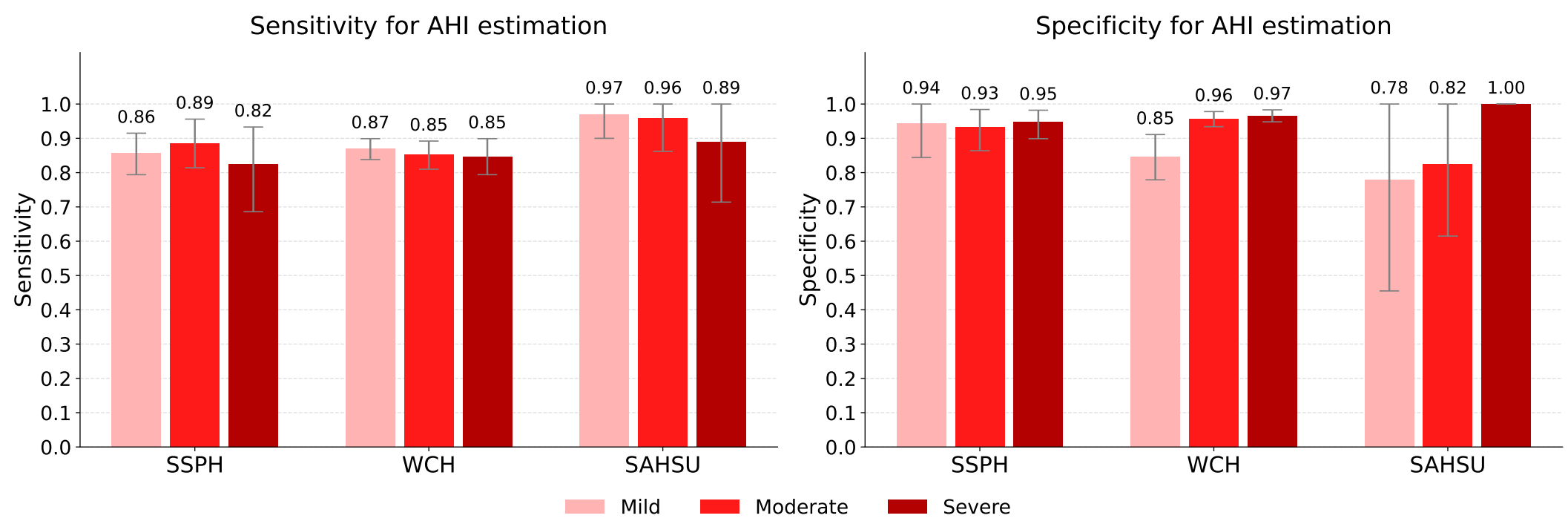

Supplementary Figure 3: Performance of BCGNet at standard AHI cutoffs. AHI cutoffs of 5, 15, and 30 were used to classify mild, moderate, and severe cases, respectively. Performance is reported for the internal set (SSPH), WCH, SAHSU, and the external cohort (WCH and SAHSU).

The clinical viability of BCGNet is further substantiated by its performance at the standard AHI cutoffs of 5, 15, and 30 events/hr (Supplementary Figure 3). The BCGNet exhibited consistently high sensitivity and specificity across all thresholds and cohorts. For instance, specificity for severe OSA (AHI > 30) was exceptional, reaching 0.95 (SSPH), 0.97 (WCH), and a perfect 1.00 (SAHSU), indicating a near-zero false positive rate for severe diagnoses. Concurrently, sensitivity for detecting moderate-to-severe disease remained high, affirming that BCGNet is a powerful tool for screening and severity assessment in clinical practice.

Supplementary Table 4: Sensitivity and specificity of BCGNet on 3-class sleep staging.

Metrics	SSPH	WCH	SAHSU	External Cohorts
Wake-Sens	0.917 (0.904,0.930)	0.898 (0.888,0.907)	0.800 (0.769,0.833)	0.889 (0.879,0.897)
NREM-Sens	0.938 (0.930,0.946)	0.940 (0.935,0.943)	0.928 (0.911,0.944)	0.938 (0.935,0.942)
REM-Sens	0.865 (0.838,0.888)	0.869 (0.857,0.879)	0.808 (0.754,0.855)	0.862 (0.851,0.873)
Wake-Spec	0.972 (0.965,0.977)	0.973 (0.970,0.975)	0.968 (0.956,0.979)	0.972 (0.969,0.975)
NREM-Spec	0.907 (0.895,0.918)	0.897 (0.890,0.904)	0.826 (0.798,0.851)	0.890 (0.882,0.896)
REM-Spec	0.978 (0.974,0.981)	0.978 (0.976,0.979)	0.967 (0.959,0.974)	0.977 (0.975,0.978)

Sens = sensitivity; Spec = specificity. Performance is reported for the internal cohort (SSPH), WCH, SAHSU, and the external cohort (WCH and SAHSU).

Supplementary Table 5: Sensitivity and specificity of BCGNet on 4-class sleep staging.

Metrics	SSPH	WCH	SAHSU	External Cohorts
Wake-Sens	0.912 (0.897, 0.925)	0.903 (0.893, 0.912)	0.785 (0.746, 0.824)	0.891 (0.881,0.901)
Light-Sens	0.838 (0.824, 0.853)	0.797 (0.790, 0.805)	0.847 (0.826, 0.869)	0.801 (0.795,0.808)
Deep-Sens	0.662 (0.617, 0.709)	0.867 (0.828, 0.903)	0.611 (0.554, 0.665)	0.732 (0.693,0.772)
REM-Sens	0.874 (0.847, 0.897)	0.870 (0.858, 0.881)	0.822 (0.775, 0.864)	0.865 (0.853,0.877)
Wake-Spec	0.972 (0.966, 0.977)	0.971 (0.968, 0.974)	0.966 (0.952, 0.979)	0.971 (0.968,0.974)
Light-Spec	0.860 (0.844, 0.874)	0.902 (0.894, 0.908)	0.775 (0.746, 0.803)	0.886 (0.878,0.893)
Deep-Spec	0.954 (0.947, 0.962)	0.919 (0.916, 0.923)	0.970 (0.962, 0.976)	0.924 (0.920,0.927)
REM-Spec	0.975 (0.971, 0.978)	0.976 (0.974, 0.978)	0.966 (0.957, 0.974)	0.975 (0.973,0.977)

Sens = sensitivity; Spec = specificity. Performance is reported for the internal cohort (SSPH), WCH, SAHSU, and the external cohort (WCH and SAHSU).

Supplementary Table 4, 5, and 6 show the performances of BCGNet in 3-, 4-, and 5- sleep staging using sensitivity and specificity. The results in 3-class staging show strong performance of BCGNet

Supplementary Table 6: Sensitivity and specificity of BCGNet on 5-class sleep staging.

Metrics	SSPH	WCH	SAHSU	External Cohorts
Wake-Sens	0.917 (0.902, 0.930)	0.913 (0.905, 0.921)	0.806 (0.771, 0.838)	0.903 (0.894,0.910)
N1-Sens	0.542 (0.504, 0.580)	0.444 (0.428, 0.460)	0.441 (0.392, 0.492)	0.444 (0.429,0.459)
N2-Sens	0.747 (0.727, 0.764)	0.726 (0.715, 0.737)	0.752 (0.725, 0.778)	0.729 (0.719,0.738)
N3-Sens	0.724 (0.673, 0.772)	0.816 (0.763, 0.863)	0.650 (0.576, 0.722)	0.729 (0.684,0.775)
REM-Sens	0.890 (0.864, 0.911)	0.894 (0.883, 0.904)	0.853 (0.807, 0.895)	0.890 (0.879,0.900)
Wake-Spec	0.974 (0.969, 0.978)	0.970 (0.966, 0.973)	0.963 (0.948, 0.977)	0.969 (0.966,0.972)
N1-Spec	0.937 (0.928, 0.944)	0.943 (0.939, 0.946)	0.917 (0.903, 0.930)	0.940 (0.936,0.943)
N2-Spec	0.883 (0.870, 0.895)	0.849 (0.842, 0.857)	0.856 (0.836, 0.877)	0.850 (0.842,0.858)
N3-Spec	0.951 (0.943, 0.959)	0.928 (0.923, 0.932)	0.960 (0.952, 0.968)	0.930 (0.926,0.935)
REM-Spec	0.973 (0.969, 0.977)	0.974 (0.972, 0.976)	0.962 (0.954, 0.969)	0.973 (0.971,0.975)

Sens = sensitivity; Spec = specificity. Performance is reported for the internal cohort (SSPH), WCH, SAHSU, and the external cohort (WCH and SAHSU).

across three sleep stages (Wake, NREM, and REM). Its high sensitivity and specificity values, particularly for NREM and REM, indicate reliable sleep staging, although there is a slight dip in performance for the SAHSU cohort. For the 4-class task, BCGNet achieved consistently high wake and REM sensitivity and specificity across all three cohorts, with slightly reduced sensitivity in the deep stage, particularly in SAHSU. In terms of the 5-class task, performance remained strong for wake, N2, N3, and REM, but sensitivity for N1 was markedly lower across all datasets, consistent with the known difficulty of distinguishing stage N1. Overall, the model demonstrated robust generalizability across cohorts with stable specificity.

High agreement in sleep continuity and architecture parameter estimation

Beyond sleep staging and AHI3% estimation, we validated BCGNet's capacity to derive key parameters of sleep continuity and architecture. We conducted Bland-Altman analyses to compare BCGNet-predicted values against PSG-derived ground truth for five standard metrics: Total Sleep Time (TST), Sleep Onset Latency (SOL), Wake After Sleep Onset (WASO), Sleep Efficiency (SE), and REM Latency (REML). The results, presented in Supplementary Figure 4, reveal a remarkable level of agreement across all three independent cohorts.

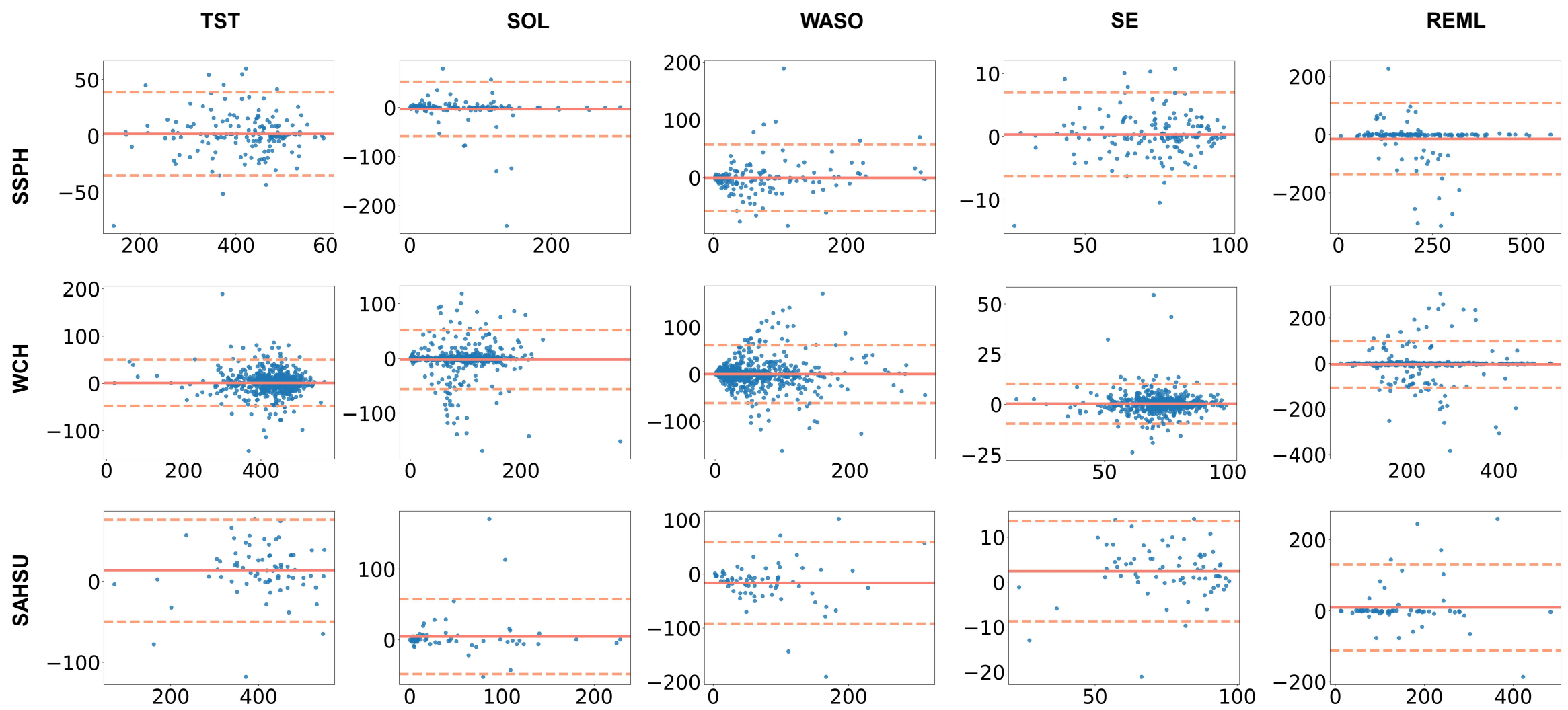

Supplementary Figure 4: Bland-Altman plots for overnight sleep continuity and architecture parameter estimation. This figure illustrates the agreement between BCGNet predicted values and ground-truth expert annotations for five sleep continuity and architecture parameters (TST, SOL, WASO, SE, REML) in the cohorts: SSPH, WCH, and SAHSU. Each panel shows the difference between predicted and true values against their mean. The solid red line represents the mean bias, and the dashed red lines indicate the 95% LoA.

For all five parameters, the mean bias (solid red line) remained near zero for most cases, suggesting that the BCGNet generally does not exhibit a strong systematic over- or under-estimation. Furthermore, the 95% limits of agreement (dashed red lines) were narrow, demonstrating strong concordance at the individual subject level. These results underscore BCGNet's potential as a reliable and comprehensive tool for sleep analysis, capable of capturing nuanced aspects of sleep quality and structure from BCG signals alone.

Ablation study on external cohorts

Supplementary Table 7: Ablation study on sleep staging and AHI3% estimation in the external cohorts.

Method	4-class sleep staging		AHI3% estimation	
	**Cohen's κ**	**F1**	**Pearson's r**	**F1**
WCH Cohort				
BCG-based	0.646 (0.636, 0.655)	0.644 (0.634, 0.653)	0.947 (0.933, 0.959)	0.680 (0.639, 0.717)
PSG-based (*w/o* fine-tuning)	0.733 (0.725, 0.741)	0.707 (0.697, 0.715)	0.952 (0.940, 0.961)	0.678 (0.639, 0.717)
BCGNet (*w/o* BCG-specific channels)	0.733 (0.725, 0.740)	0.706 (0.696, 0.714)	0.952 (0.939, 0.963)	0.690 (0.652, 0.726)
**BCGNet (proposed)**	**0.736 (0.728, 0.743)**	**0.710 (0.700, 0.718)**	**0.959 (0.949, 0.968)**	**0.703 (0.668, 0.741)**
SAHSU Cohort				
BCG-based	0.546 (0.513, 0.581)	0.665 (0.641, 0.693)	0.933 (0.887, 0.972)	0.679 (0.517, 0.813)
PSG-based (*w/o* fine-tuning)	0.687 (0.655, 0.715)	0.771 (0.748, 0.790)	0.935 (0.894, 0.972)	**0.766 (0.595, 0.886)**
BCGNet (*w/o* BCG-specific channels)	**0.698 (0.670, 0.724)**	0.779 (0.758, 0.797)	0.946 (0.908, 0.976)	0.762 (0.598, 0.898)
**BCGNet (proposed)**	0.697 (0.666, 0.725)	**0.781 (0.759, 0.800)**	**0.952 (0.920, 0.981)**	0.764 (0.595, 0.892)

"*w/o*" stands for "without". The results are based on the external WCH and SAHSU cohorts. The bolded values indicate the highest performance for each task.

Supplementary Table 7 summarizes the ablation results on external cohorts, extending our analysis to isolate the specific contribution of the multi-frequency BCG channels. In the WCH dataset, incorporating the BCG-specific channels (Proposed BCGNet) yielded consistent improvements over both the baseline BCG-based model and the variant relying solely on transferred channels (*w/o* BCG-specific channels), affirming the complementary value of raw signal features in large diverse cohorts. In the smaller SAHSU cohort, while the PSG-based model without fine-tuning showed an unexpectedly high F1 score for AHI3% estimation due to sample size variance, the proposed BCGNet maintained high stability, matching the highest staging agreement ($\kappa = 0.697$ vs 0.698) and yielding the highest staging F1 score (0.781). Overall, these results quantitatively justify our architectural choice: the Dynamic Feature Fusion effectively leverages both abstracted physiological concepts and high-fidelity raw BCG components to maintain robustness across heterogeneous clinical environments.

Adaptive Temporal Down-sampling Strategy

To effectively fuse physiological signals recorded at vastly different sampling rates, ranging from 2 Hz for respiration to 400 Hz for snore audio, we designed a modality-specific input processing pipeline. The goal was to preserve the high-frequency morphological details of the raw signals while ensuring that the extracted feature vectors from all encoders possess consistent temporal dimensions for the subsequent *Dynamic Feature Fusion* module.

As detailed in Supplementary Table 8, the token length (L_{in}) for each channel is determined by the product of the task-specific window duration (T) and the channel's native sampling rate (f_s): $L_{in} = T \times f_s$. For the AHI3% estimation task ($T = 1$s) and the Sleep Staging task ($T = 30$s), this results in input vectors of varying lengths. To align these inputs, the 1D ResNet feature extractor

Supplementary Table 8: Detailed configuration of each signal modality.

Signal Modality	Sampling Rate (Hz)	AHI3% Estimation (Token duration = 1 s)		Sleep Staging (Token duration = 30 s)	
		**Token Length**	**Strides (5 Blocks)**	**Token Length**	**Strides (5 Blocks)**
Transferred Channels					
PSG/BCG Resp/Heart	4	4	[1, 1, 1, 2, 2]	120	[2, 2, 2, 3, 5]
BCG-Specific Channels					
BCG Respiration	2	2	[1, 1, 1, 1, 2]	60	[1, 2, 2, 3, 5]
BCG Heartbeat	50	50	[1, 1, 2, 5, 5]	1500	[2, 3, 5, 5, 5]
BCG Snore	400	400	[2, 4, 4, 5, 5]	12000	[4, 4, 4, 5, 15]

The "Strides" column lists the stride values applied sequentially in the five blocks of the ResNet encoder. The distinct stride combinations ensure that despite the varying input lengths (Token Length), the output feature maps are down-sampled to a compatible temporal resolution for feature fusion.

for each channel employs a customized configuration of convolutional strides across its five blocks. The product of the strides approximates the input length, effectively down-sampling the temporal dimension to a unified scale.

Model Complexity and Inference Efficiency

To assess the feasibility of deploying BCGNet for daily home monitoring applications, we evaluated the model's computational complexity and inference efficiency. The evaluation encompassed four key metrics: total number of trainable parameters, model size, computational complexity, and inference latency.

The benchmark was conducted under realistic inference scenarios representing an entire night of sleep. For the Sleep Staging task, the model processed a continuous 12-hour input sequence. For the AHI3% estimation task, we adhered to the standard inference protocol detailed in Section 4.4, where a 30-minute contextual window is slid across the entire night. Using a 6-minute stride, a 12-hour recording generates approximately 116 segments. We evaluated the computational cost for both a single 30-minute segment (single forward pass) and the batched whole-night sequence. Inference latency was measured on an NVIDIA H20 GPU and averaged over 100 independent runs to ensure reliability.

As presented in Supplementary Table 9, BCGNet demonstrates high computational efficiency. The model footprint is compact (approximately 248 MB with 38.14 million parameters). Notably, the inference latency for processing a full 12-hour sleep recording for staging is highly optimized at 31.01 ms. For AHI estimation, processing a single 30-minute window takes 17.71 ms, and sequentially processing the entire 12-hour night via sliding windows takes under one second (943.77 ms). These metrics confirm that BCGNet is exceptionally well-suited for scalable, real-world deployment with minimal computational bottlenecks.

Supplementary Table 9: Model complexity and inference efficiency of BCGNet.

Metric	Sleep Staging	AHI3% Estimation	
	(12-hour continuous input)	(Single 30-min window)	(12-hour continuous input)
Total Trainable Parameters	38.14 M	38.14 M	38.14 M
Model Size	248.00 MB	248.12 MB	248.12 MB
Computational Complexity	80.27 GFLOPs	108.11 GFLOPs	12.54 TFLOPs
Inference Latency	31.01 ms	17.71 ms	943.77 ms

Note on metrics: **M** = Million (count of trainable weights and biases); **MB** = Megabytes (physical memory footprint of the saved model file); **GFLOPs / TFLOPs** = Giga / Tera Floating-point Operations (indicating the computational workload); **ms** = milliseconds (inference time). Latency measurements were averaged over 100 runs on an NVIDIA H20 GPU. The whole-night AHI estimation simulates processing a 12-hour recording using a 30-minute sliding window with a 6-minute stride.

Training Dynamics and Learning Curves

To illustrate the convergence behavior and stability of BCGNet during the training phase, we present the learning curves for both the AHI3% estimation task (Supplementary Figure 5) and the Sleep Staging task (Supplementary Figure 6).

For both tasks, the final model checkpoints (indicated by the red dashed vertical lines) were selected based on the peak of the primary validation metric—Validation F1 score for AHI estimation and Validation Cohen's κ for sleep staging—rather than the absolute minimum of the validation loss. In complex physiological signal classification, the continuous loss function (which measures probabilistic confidence) may experience a slight rebound in later epochs as the model evaluates difficult examples. However, the actual discrete decision boundaries, as captured by F1 and κ, often continue to optimize. Thus, selecting the model based on these primary target metrics ensures superior and more robust performance in real-world clinical evaluations.

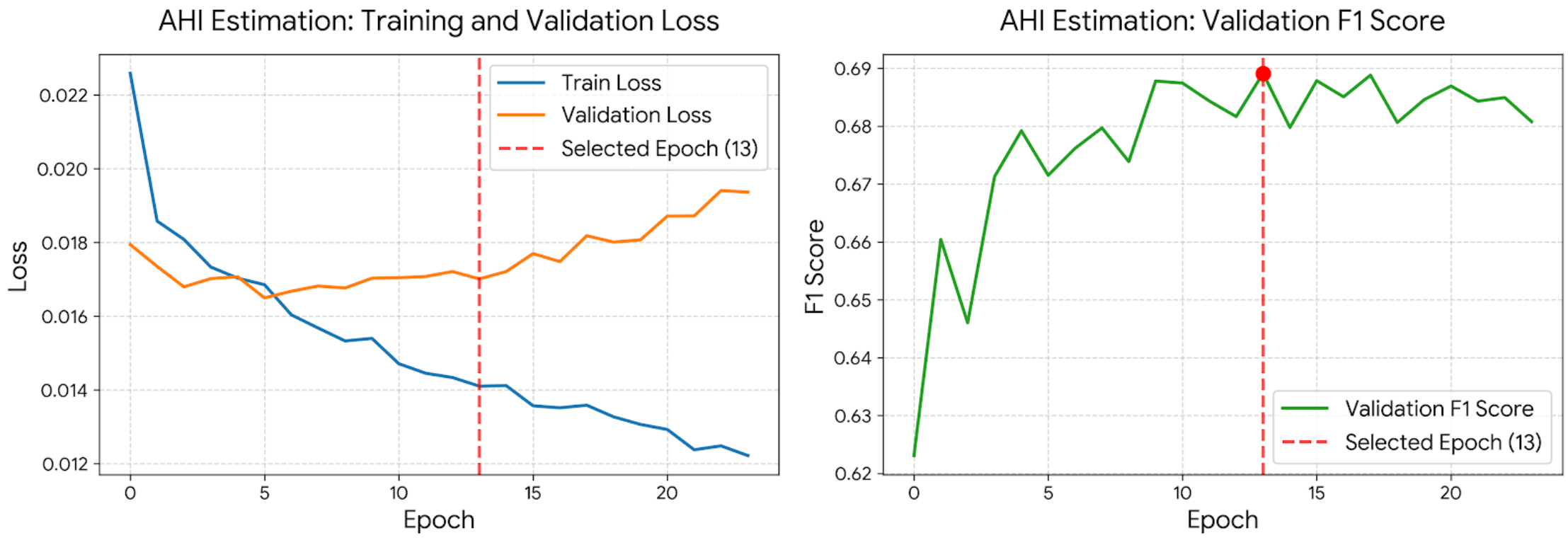

Supplementary Figure 5: **Learning curves for the** AHI3% **estimation task.** The left panel displays the training and validation loss over epochs, while the right panel shows the progression of the validation F1 score. The red dashed line marks the selected optimal model checkpoint corresponding to the highest validation F1 score.

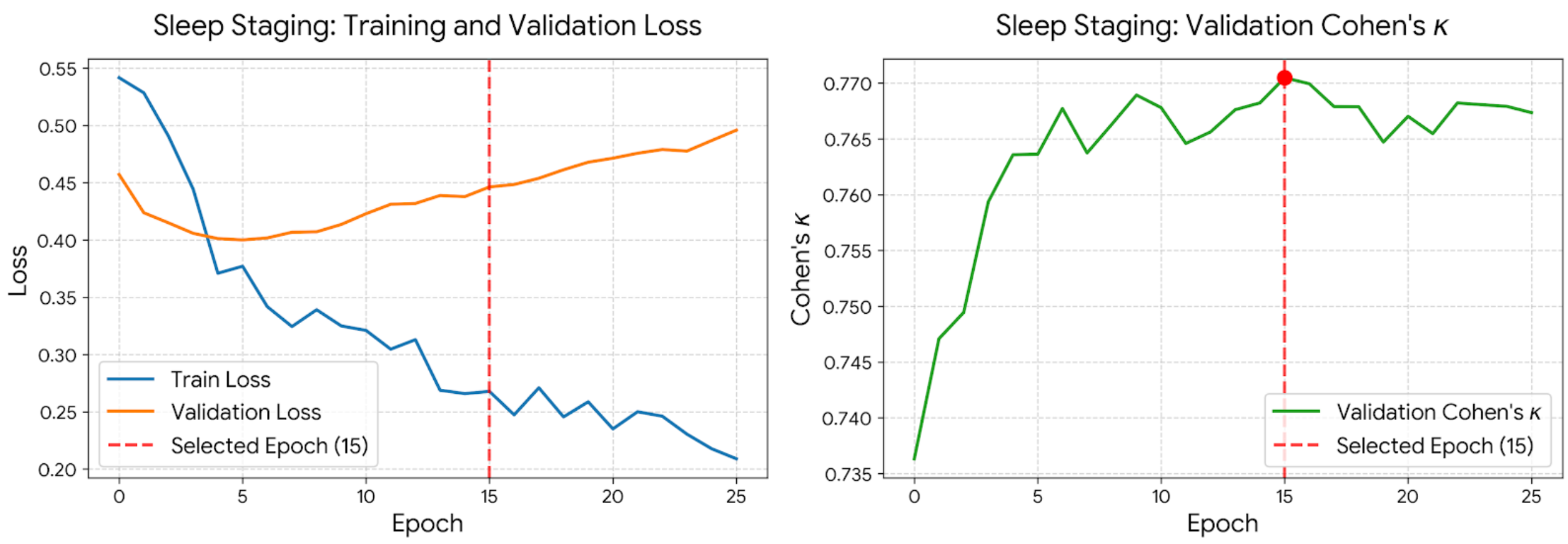

Supplementary Figure 6: **Learning curves for the Sleep Staging task.** The left panel illustrates the training and validation loss, and the right panel presents the validation Cohen's κ. The red dashed line denotes the selected checkpoint based on the peak validation κ.
